# Gene Expression as a Guide to the Development of Novel Therapies in Primary Glomerular Diseases

**DOI:** 10.3390/jcm10112262

**Published:** 2021-05-24

**Authors:** Panagiotis Garantziotis, Stavros A. P. Doumas, Ioannis Boletis, Eleni Frangou

**Affiliations:** 1 Department of Clinical Immunology and Rheumatology, Medical University Hannover, 30625 Hannover, Germany; garantziotis.p@gmail.com; 2 Lab of Autoimmunity and Inflammation, Biomedical Research Foundation of the Academy of Athens, 11527 Athens, Greece; sap.doumas@gmail.com; 3 Department of Nephrology and Renal Transplantation, University of Athens Medical School, Laiko Hospital, 11527 Athens, Greece; laikneph@laiko.gr; 4 Department of Nephrology, Limassol General Hospital, 3304 Limassol, Cyprus; 5 Medical School, University of Nicosia, 2417 Nicosia, Cyprus

**Keywords:** primary glomerulonephritis, focal segmental glomerulosclerosis, minimal change disease, IgA nephropathy, membranous nephropathy, thin basement membrane nephropathy, gene expression, computational systems biology, drug discovery and development, drug repurposing

## Abstract

Despite improvements in understanding the pathogenic mechanisms of primary glomerular diseases, therapy still remains nonspecific. We sought to identify novel therapies targeting kidney-intrinsic injury of distinct primary glomerulonephritides through computational systems biology approaches. We defined the unique transcriptional landscape within kidneys from patients with focal segmental glomerulosclerosis (FSGS), minimal change disease (MCD), immunoglobulin A nephropathy (IgAN), membranous nephropathy (MN) and thin basement membrane nephropathy (TBMN). Differentially expressed genes were functionally annotated with enrichment analysis, and distinct biological processes and pathways implicated in each primary glomerular disease were uncovered. Finally, we identified novel drugs and small-molecule compounds that may reverse each glomerulonephritis phenotype, suggesting they should be further tested as precise therapy in primary glomerular diseases.

## 1. Introduction

Primary glomerulonephritides encompass a heterogeneous group of glomerular diseases characterized by abnormal activation of innate and/or adaptive immune responses due to kidney-intrinsic factors [1]. Although relatively rare, they represent the most common cause of end-stage renal disease in young adults and are associated with increased morbidity, mortality and healthcare costs [2]. Current clinicopathological classification includes minimal change disease (MCD), focal segmental glomerulosclerosis (FSGS), membranous nephropathy (MN), immunoglobulin and complement-mediated glomerular diseases with a membranoproliferative glomerular pattern (MPGN), immunoglobulin A nephropathy (IgAN) and thin basement membrane nephropathy (TBMN) [1]. Despite improvements in understanding the underlying pathogenic mechanism of each glomerular disease, therapy still remains nonspecific and includes general supportive measures coupled with immunosuppression [3,4]. Disease-specific therapy targeting kidney-intrinsic injury still remains a major challenge in nephrology.

Recent advances in omics technologies have provided insights into the molecular mechanisms underlying complex traits, such as glomerular diseases. Gene expression represents the intermediate phenotype between genetic variation and disease phenotypic variation and thus may inform about genetic and environmental effects on cells and tissues. Specifically, the comparison of gene expression variation between distinct conditions can delineate transcriptional differences and specific molecular pathways [5]. To this end, high-throughput genome-wide gene expression studies have described the transcriptome of the peripheral blood and kidneys of animal models and patients with glomerular diseases and uncovered molecular pathways implicated in their pathogenesis [6]. However, gene expression patterns unique to each primary glomerular disease remain to be defined.

Computational systems biology combines knowledge-driven experimental data with simulation-based analyses and tests hypotheses with in silico experiments. This provides a powerful tool to understand complex biological processes and identify novel drugs or drugs to be repurposed [7]. The Connectivity Map (CMap) project was the first approach that provided genome-wide gene expression responses from 4 human cell lines treated with 1 out of 1309 FDA-approved drugs or small-molecule compounds at different doses [8]. The NIH Library of Integrated Network-based Cell-Signatures (LINCS) program expanded the CMap project to include over a million signatures through the use of the L1000 high-throughput transcriptomic technology; it identified gene expression alterations (before and after treatment) of more than 60 human cell lines with more than 20,000 drugs/small-molecule compounds [9,10]. By the use of a multivariate method to compute signatures, the LINCS L1000 Characteristic Direction Signatures Search engine (L1000CDS^2^) further prioritized thousands of small-molecule signatures and their pairwise combinations, predicted to either mimic or reverse the gene expression signature of a disease or condition [11]. Recently, connectivity mapping identified BI-2536 as a potential drug to treat diabetic nephropathy [12].

Herein, we employed the Nephroseq classic v4 web-based analysis engine [13], a platform for integrative data mining of comprehensive kidney disease gene expression datasets, and identified datasets that compared kidney gene expression patterns from patients with primary glomerular diseases vs. healthy individuals. We defined the unique transcriptional landscape of FSGS, MCD, IgAN, MN and TBMN through the comparison of kidney gene expression patterns between the yielded datasets. Differentially expressed genes were functionally annotated with enrichment analysis, and distinct biological processes and pathways implicated in each primary glomerular disease were established. Finally, using the L1000CDS^2^ engine [11], we identified putative novel drugs or small-molecule compounds that may reverse each glomerulonephritis phenotype, suggesting they should be further tested as precise therapy in primary glomerular diseases.

## 2. Materials and Methods

### 2.1. Search Strategy

To identify publicly available gene expression profiles of human glomeruli from patients with primary glomerular diseases, we used the Nephroseq classic v4 web-based engine [13]. Gene expression datasets generated using the Affymetrix Microarray Technology were only included in the study. Specifically, the keywords “human”, “kidney”, “glomerulus”, “microarray” and “affymetrix” were used in the engine to search for gene expression patterns of diseases. Expression profiles from minimal change disease (MCD), focal segmental glomerulosclerosis (FSGS), membranous nephropathy (MN), immunoglobulin A nephropathy (IgAN) and thin basement membrane nephropathy (TBMN), when compared to expression profiles from healthy kidneys, were yielded. We applied a *p*-value cutoff of 0.05, as provided by the Nephroseq classic v4 web-based engine, to define the statistically significant differentially expressed genes (DEGs). All DEGs that demonstrated statistical significance, irrespective of their fold-change, were included in the analysis. Comparison between DEGs was performed with Venny^2.1^ [14] and InteractiVenn [15].

### 2.2. Functional Enrichment Analysis and Drug Prediction

Enrichment analysis of DEGs was performed using g:Profiler [16]. The visualization of enrichment analysis was carried out using the GraphPad Prism version 5.0 [17]. To identify compounds that efficiently reverse the disease-specific transcriptional signatures, the L1000CDS^2^ engine was used [11]. To identify the possible mechanism of action, disease target, side effects and FDA approval of the drugs/small molecules, we used the Large-Scale Visualization of Drug-induced Transcriptomic signatures (L1000FWD) engine [18], the DrugBank resource [19] and the FDA website.

## 3. Results

### 3.1. Computational Systems Biology Approaches Reveal Glomerulonephritis-Specific Gene Signatures

We initially employed the Nephroseq classic v4 web-based analysis engine [13] to identify datasets of kidney gene expression patterns from patients with primary glomerular diseases and healthy individuals. Therefore, we collected previously published datasets that compared kidney gene expression from patients with focal segmental glomerulosclerosis (FSGS) (a total of 39 patients) [20,21], minimal change disease (MCD) (a total of 21 patients) [20,21], IgA nephropathy (IgAN) (a total of 54 patients) [21,22], membranous nephropathy (MN) (21 patients) [21] or thin basement membrane nephropathy (TBMN) (3 patients) [21], with a total of 36 healthy individuals. Clinical parameters and demographics of patients and healthy individuals are presented in Table S1. 

**Figure 1:**
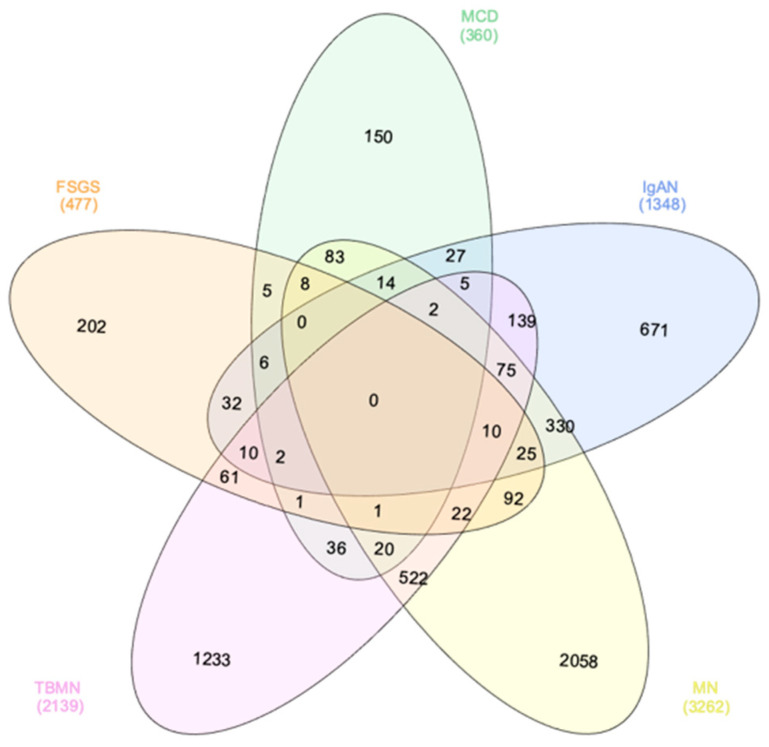
Glomerulonephritis-specific differentially expressed genes. Gene expression patterns between primary glomerular diseases were compared. Differentially expressed genes (DEGs) that were exclusively identified in one glomerular disease (and not in others) defined the glomerulonephritis-specific gene signatures. FSGS—focal segmental glomerulosclerosis; MCD—minimal change disease; IgAN—immunoglobulin A nephropathy; MN—membranous nephropathy; TBMN—thin basement membrane nephropathy.

To define the unique transcriptional landscape of each primary glomerular disease, we compared gene expression patterns between the yielded datasets. Differentially expressed genes (DEGs) that were exclusively identified in one glomerular disease (and not in others) defined the glomerulonephritis-specific gene signature. Accordingly, 202, 150, 671, 2058 and 1233 DEGs defined the FSGS-specific, MCD-specific, IgAN-specific, MN-specific and TBMN-specific gene signature, respectively, suggesting a unique pathogenic involvement highly specific to the respective glomerular disease. Interestingly, no common DEGs across all diseases were identified (Figure 1, Table S2). These data demonstrate that gene expression patterns within kidneys in primary glomerular diseases are deregulated in a glomerulonephritis-specific manner. Experimental validation of differential transcripts and their functionality in kidneys is further needed.

### 3.2. Unique Biological Processes and Pathways Are Implicated in Distinct Primary Glomerular Diseases

**Figure 2:**
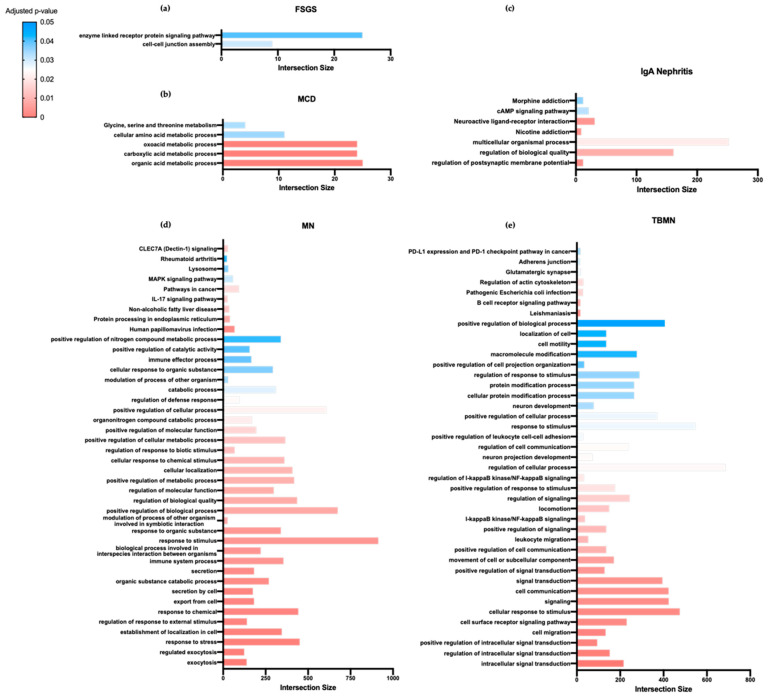
Functional enrichment analysis of glomerulonephritis-specific differentially expressed genes (DEGs) reveals the involvement of unique biological processes and pathways in distinct primary glomerular diseases. Bar-plot diagrams demonstrating functional enrichment analysis of DEGs from the (**a**) FSGS-specific gene signature; (**b**) MCD-specific gene signature; (**c**) IgAN-specific gene signature; (**d**) MN-specific gene signature; (**e**) TBMN-specific gene signature. Intersection size demonstrates the number of genes identified in each enriched term. Colors denote adjusted p-values of terms as defined by the color-coding heatmap. FSGS—Focal Segmental Glomerulosclerosis; MCD—Minimal Change Disease; IgAN—Immunoglobulin A nephropathy; MN—Membranous nephropathy; TBMN—Thin Basement Membrane Nephropathy.

Next, to reveal altered molecular pathways between primary glomerular diseases, glomerulonephritis-specific DEGs were functionally interpreted through enrichment analysis using g:Profiler [16]. To this end, the FSGS-specific gene signature was functionally enriched in gene ontology (GO) biological processes linked to cell–cell junction assembly and the enzyme-linked receptor protein signaling pathway. Similarly, the MCD-specific gene signature was functionally enriched in glycine serine threonine metabolism; the IgAN-specific gene signature was enriched in regulation of postsynaptic membrane potential and the cAMP signaling pathway; the MN-specific gene signature was enriched in IL-17 and MAPK signaling pathways, whereas the TBMN-specific gene signature was enriched in pathways linked to B-cell receptor signaling and regulation of actin cytoskeleton (Figure 2). Collectively, these data demonstrate the involvement of unique biological processes and pathways in distinct glomerular diseases, suggesting that these processes and pathways may represent disease-specific novel therapeutic targets.

To identify putative novel drugs or small-molecule compounds that may reverse each glomerulonephritis phenotype, we used the L1000 Characteristic Direction Signature Search Engine (L1000CDS^2^) [16]. The L1000CDS^2^ was queried with upregulated and downregulated genes from each glomerular disease to prioritize matching signatures obtained from more than 20,000 drug and small-molecule treatments of multiple human cell lines. Queries generated predictions for the top 50 molecules that could reverse the input of each glomerular disease (Table S3). 

**Figure 3:**
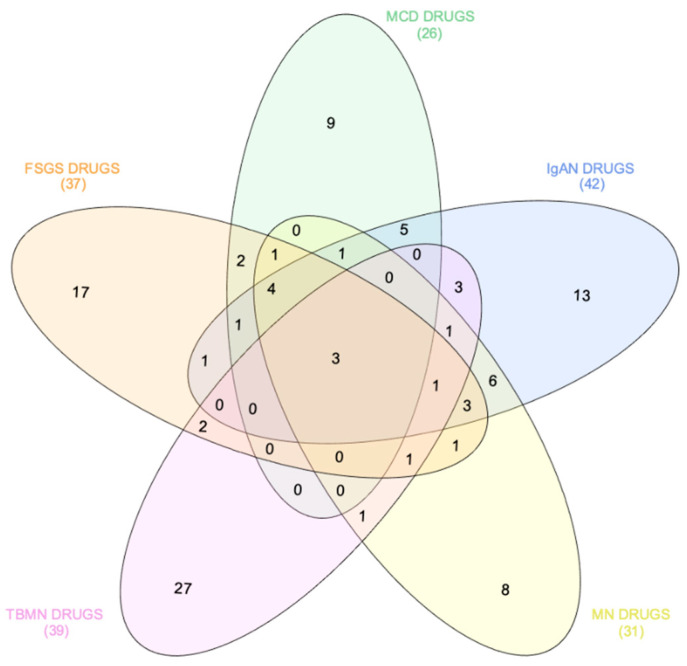
Drugs and small-molecule compounds predicted to reverse gene expression patterns of primary glomerulonephritides in a disease-specific manner. The comparison of prioritized drugs/small-molecule compounds that may reverse gene expression patterns from primary glomerular diseases revealed glomerulonephritis-specific drugs and drugs that may reverse gene expression patterns from all glomerulonephritides. FSGS—focal segmental glomerulosclerosis; MCD—minimal change disease; IgAN—immunoglobulin A nephropathy; MN—membranous nephropathy; TBMN—thin basement membrane nephropathy.

To identify glomerulonephritis-specific novel drugs or small-molecule compounds, we compared the top 50 drug signatures between the 5 glomerular diseases. Drugs or small-molecule compounds that were predicted to reverse only one glomerulonephritis-specific gene signature and not others defined the glomerulonephritis-specific drug signature. Thus, 17, 9, 13, 8 and 27 drugs/small-molecule compounds were predicted to reverse the FSGS-specific, MCD-specific, IgAN-specific, MN-specific and TBMN-specific gene signature, respectively (Figure 3, Table 1). 

Interestingly, PD-0325901 (an investigational MEK inhibitor [23]), PD-184352 (an investigational MAPK inhibitor [24]) and KU 0060648 trihydrochloride were predicted to reverse gene expression patterns of all glomerular diseases (Table 2).

Finally, the potential mechanism of action of identified drugs and small molecules, the disease they may target, their possible side effects and their possible FDA approval were identified through the L1000FWD engine [18], the DrugBank resource [19] and the FDA website (Table 3).

Collectively, our data reveal novel, not previously identified, drugs and small-molecule compounds that may reverse the phenotype of primary glomerular diseases in a disease-specific manner. Further investigation is called for to define the therapeutic implications of our findings.

## 4. Discussion

Herein, we employed the Nephroseq classic v4 web-based analysis engine and identified gene expression datasets within kidneys from patients with primary glomerular diseases vs. healthy individuals. The comparison of gene expression patterns defined the FSGS-specific, MCD-specific, IgAN-specific, MN-specific and TBMN-specific gene signatures. Functional enrichment analysis of differentially expressed genes identified distinct biological processes and pathways implicated in each primary glomerular disease. Finally, by the use of computational systems biology resources, we revealed novel, not previously identified, drugs and small-molecule compounds that may reverse the phenotype of primary glomerular diseases in a glomerulonephritis-specific manner.

We elected to use the Nephroseq classic v4 web-based analysis engine that gives access to genome-wide gene expression datasets generated by renal researchers [13]. We yielded datasets generated from hybridization-based analyses of human kidneys from patients with primary glomerular diseases when compared to healthy kidneys. Specifically, we obtained datasets for MCD, FSGS, MN, IgAN and TBMN. Although these diseases are characterized by distinct pathogenic mechanisms and express distinct manifestations, therapy is common and includes general measures to reduce proteinuria and manage hypertension, edema, hyperlipidemia and hypercoagulability, coupled with immunosuppression, such as steroids, alkylating agents, mycophenolate mofetil, calcineurin inhibitors and others) [3,4,25]. To reveal glomerulonephritis-specific processes and pathways implicated in each glomerular disease that could serve as glomerulonephritis-specific targets, we compared the kidney transcriptome between the yielded primary glomerular diseases. We uncovered DEGs exclusively expressed in each glomerular disease that defined each glomerulonephritis-specific gene signature. Gene signatures were further interpreted with functional enrichment analysis, by which delineated processes and pathways unique to each glomerular disease were uncovered.

FSGS and MCD are primary podocytopathies that underlie idiopathic nephrotic syndrome. FSGS is a heterogeneous disease that represents a common phenotypic expression of different clinical and histological syndromes caused by distinct causes, including mutations in podocyte components, circulating factors, viral infections, toxicities and maladaptive responses [26,27,28]. Specifically, the pathogenesis involves a factor disturbing podocyte function and permeability and a T-cell abnormality leading to cytokine production [29,30]. Cardiotrophin-like cytokine-1 (CLC-1) and soluble urokinase plasminogen activator surface receptor (suPAR) are implicated in the pathogenesis of FSGS [31,32]. MCD represents the most common cause of nephritic syndrome in children. Underlying causes may include Hodgkin disease, atopy, exposure to allergens and nonsteroidal anti-inflammatory drugs [33,34,35]. In MCD, deregulated T-cell function can be attributed to altered expression of NF-κB and NFRKB [36,37]. Our data demonstrate that gene expression deregulation within kidneys of human FSGS is associated with biological processes linked to cell–cell junction assembly and the enzyme-linked receptor protein signaling pathway. Gene expression deregulation within kidneys from human MCD is linked to glycine, serine and threonine metabolism. These suggest that the identified processes and pathways may represent targeted therapies for FSGS or MCD.

The current model of IgAN pathogenesis involves a multiple-hit hypothesis model. Genetic and environmental factors predispose individuals to abnormal immune responses to pathogens. Mucosal-derived pathogen-associated molecular patterns induce polyclonal lymphocyte proliferation in a TLR-mediated manner. IgA-secreting plasma cells migrate to mucosa, releasing dimeric IgA into the lumen, whereas misdirected cells are released to systemic circulation, secreting poorly O-galactosylated IgA1. Circulating galactose-deficient IgA1 forms immune complexes with specific antibodies, resulting in immune-complex deposition or in situ formation in the mesangium, inducing mesangial cell proliferation and increased production of mesangial matrix [38,39]. Despite improvements in understanding the pathogenesis of IgAN, this multiple-hit hypothesis model does not explain kidney injury associated with mesangial IgA deposition. Our analysis demonstrates that within kidneys of patients with IgAN, gene expression deregulation is associated with neuroactive ligand-receptor interaction and the cAMP signaling pathways, suggesting they could represent targets of kidney injury in IgAN.

MN is an autoimmune disease characterized by the formation of immune deposits and complement-mediated proteinuria [40]. Epitopes and antigens, such as phospholipase A2 receptor (PLA2R) [41], thrombospondin domain-containing 7A (THSD7A) [42] and neutral endopeptidase [43], have been identified. The role of complement activation mediating podocyte injury in MN is well established [44,45]. Recently, the altered T_H_17/T_reg_ ratio was suggested as a mechanism involved in the pathogenesis of idiopathic MN [46]. In agreement, we reveal that within kidneys of patients with MN, the transcriptome deregulation is functionally enriched in IL17 signaling pathways. We also uncover the role of CLEC7A (dectin) and MAPK signaling pathway, suggesting their involvement in tissue injury in idiopathic MN.

TBMN refers to familial and sporadic isolated hematuria associated with an attenuated glomerular basement membrane. It is an autosomal dominant condition caused by mutations in *COL4A3* or *COL4A4* gene [47]. In this study, we demonstrate that within kidneys from patients with TBMN, DEGs are functionally enriched in pathways of B-cell receptor signaling and regulation of actin cytoskeleton.

Next, we used the identified glomerulonephritis-specific gene signatures, and through computational systems biology approaches, we sought to discover novel drugs or drugs to be repurposed in a disease-specific manner. Specifically, we used the L1000CDS^2^ engine [11] and prioritized the top 50 molecules predicted to reverse upregulated and downregulated DEGs of each glomerular disease. Then, drug signatures of the five glomerulonephritides were compared, and glomerulonephritis-specific drugs were identified. Among other drugs, the third-generation kinase inhibitor indicated for the treatment of chronic myeloid leukemia, *ponatinib* [48], and the *JAK3 kinase inhibitor IV* [49] were predicted to specifically reverse the FSGS-specific gene signature; *pracinostat*, an oral histone deacetylase inhibitor [50] was predicted to specifically reverse the MCD-specific gene signature; *BMS-754807*, an inhibitor of insulin-like growth factor-1R/IR [51], was predicted to specifically reverse the IgAN-specific gene signature; *TCPA-1*, a dual inhibitor of STAT3 and NF-κB [52], was predicted to specifically reverse the MN-specific gene signature; and *wortmannin*, an autophagy inhibitor [53], was predicted to specifically reverse the TBMN-specific gene signature. This computational analysis prioritized novel drugs and drugs to be repurposed as glomerulonephritis-specific agents. Further investigation is ongoing to define the therapeutic implications of our findings.

Collectively, through computational systems biology approaches, we defined the FSGS-specific, MCD-specific, IgAN-specific, MN-specific and TBMN-specific gene signatures within human kidneys and identified distinct biological processes and pathways implicated in each disease. We also revealed novel, not previously identified, drugs and small-molecule compounds that may reverse the phenotype of primary glomerular diseases in a glomerulonephritis-specific manner. Experimental validation of the differential transcripts and their functionality is further needed. To this end, future studies will define the pathogenic and therapeutic implications of our findings.

## Figures and Tables

**Table 1:** Glomerulonephritis-specific drugs. Drugs and small-molecule compounds predicted to reverse the glomerulonephritides-specific gene signatures. They are presented according to their ranking and score during prioritization. Cell line tested and dose and time of treatment applied to each cell line are demonstrated in separate columns.

Specific Drugs	Rank	Score	Perturbation (Drug)	Cell Line	Dose	Time
FSGS-specific	3	0.0344	Tanespimycin	HEPG2	10.0 um	24 h
	4	0.0323	JAK3 Inhibitor VI	PC3	10.0 um	6 h
	48	0.0237	BRD-A17065207	SKB	10.0 um	24 h
	10	0.0301	Ponatinib	HEPG2	3.33 um	24 h
	11	0.0301	NVP-AUY922	HEPG2	0.37 um	24 h
	23	0.0258	BRD-K53414658	A375	10.0 um	24 h
	28	0.0258	KIN001-265	HEPG2	0.37 um	24 h
	31	0.0258	CGP-60474	MCF10A	1.11 um	24 h
	33	0.0258	GSK-1059615	BT20	10.0 um	24 h
	34	0.0258	AZD-5438	BT20	10.0 um	24 h
	36	0.0258	GSK-2126458	LNCAP	1.11 um	24 h
	38	0.0258	AZ-628	A375	3.33 um	24 h
	39	0.0237	Z-Leu3-VS	HCC515	10.0 um	24 h
	41	0.0237	CGP 71683 hydrochloride	HCC515	10.0 um	24 h
	42	0.0237	KETOROLAC TROMETHAMINE	HCC515	10.0 um	24 h
	45	0.0237	16-HYDROXYTRIPTOLIDE	HA1E	0.08 um	24 h
	46	0.0237	89671	HA1E	0.09 um	24 h
MCD-specific	11	0.0453	BRD-K13810148	MCF7	10.0 um	24 h
	16	0.0453	Pracinostat	A549	10.0 um	24 h
	18	0.0425	NORETHINDRONE	VCAP	10.0 um	24 h
	25	0.0425	5-Fluorocytosine	VCAP	10.0 um	24 h
	30	0.0397	TWS119	HCC515	10.0 um	24 h
	37	0.0397	DL-PDMP	A375	64.0 um	24 h
	42	0.0397	Taxifolin-(+/−)	VCAP	10.0 um	24 h
	44	0.0397	EI-293	PC3	10.0 um	24 h
	46	0.0397	Neratinib	HCC515	3.33 um	24 h
IgAN-specific	3	0.0296	BMS-754807	A375	10.0 um	24 h
	4	0.0296	BRD-K19295594	A375	11.1 um	24 h
	11	0.0281	BRD-K65814004	MCF7	10.0 um	24 h
	18	0.0266	DG-041	A375	40.0 um	24 h
	25	0.0258	15-Deoxy-12,14-prostaglandin J2	A375	10.0 um	24 h
	27	0.0258	Dovitinib	A375	1.11 um	24 h
	28	0.0258	Mitoxantrone	A375	0.12 um	24 h
	32	0.0251	7b-cis	A375	10.0 um	24 h
	39	0.0243	MK-0591	A375	80.0 um	24 h
	41	0.0243	Piperlongumine (HPLC)	PC3	10.0 um	24 h
	43	0.0243	S1367	MCF7	10.0 um	24 h
	46	0.0243	NVP-BEZ235	A549	0.37 um	24 h
	47	0.0243	SB590885	HT29	3.33 um	24 h
MN-specific	9	0.0183	Foretinib	HT29	10.0 um	24 h
	34	0.0151	WH-4-025	HT29	10.0 um	24 h
	13	0.0170	GDC-0980	HT29	10.0 um	24 h
	23	0.0157	TPCA-1	HT29	10.0 um	24 h
	25	0.0157	S1175	MCF7	10.0 um	24 h
	31	0.0154	BRD-K41859756	HT29	10.0 um	24 h
	41	0.0148	Palbociclib	HME1	10.0 um	24 h
	49	0.0145	Erlotinib	MCF10A	3.33 um	24 h
TBMN-specific	14	0.0243	Narciclasine	PC3	10.0 um	6 h
	10	0.0252	BRD-A93236127	MCF7	10.0 um	24 h
	12	0.0247	BRD-A62809825	HT29	10.0 um	24 h
	13	0.0247	Homoharringtonine	HT29	10.0 um	6 h
	16	0.0238	ARP 101	MCF7	10.0 um	24 h
	17	0.0228	CAY10594	HT29	20.0 um	24 h
	18	0.0228	BRD-K91370081	SKB	10.0 um	24 h
	19	0.0223	BRD-K32896438	MCF7	10.0 um	24 h
	20	0.0223	BRD-K23478508	MCF7	10.0 um	24 h
	21	0.0223	BRD-K53308430	VCAP	10.0 um	24 h
	22	0.0218	Wortmannin	PC3	10.0 um	24 h
	23	0.0218	BJM-ctd2-9	SW620	10.0 um	6 h
	24	0.0218	HY-10518	VCAP	10.0 um	24 h
	26	0.0218	BRD-K06009608	VCAP	10.0 um	24 h
	28	0.0218	LDN-193189	SKBR3	10.0 um	3 h
	50	0.0204	BRD-K92317137	PC3	10.0 um	6 h
	30	0.0213	PK-11195	HT29	160.0 um	24 h
	31	0.0213	MST- 312	PC3	11.1 um	24 h
	32	0.0213	BRD-K80786583	MCF7	10.0 um	24 h
	33	0.0213	BRD-K78122587	MCF7	10.0 um	24 h
	37	0.0213	GDC-0941	LNCAP	1.11 um	24 h
	38	0.0209	PERHEXILINE MALEATE	HT29	10.0 um	24 h
	40	0.0209	Dorsomorphin dihydrochloride	MCF7	10.0 um	24 h
	41	0.0209	BRD-U33728988	SKB	10.0 um	24 h
	44	0.0209	BRD-A80502530	MCF7	10.0 um	24 h
	45	0.0209	PHA-665752	HT29	0.04 um	24 h
	47	0.0209	JW-7-24-1	A549	10.0 um	24 h

**Table 2:** Drugs that may reverse gene expression patterns from all glomerular diseases. Drugs and small-molecule compounds are presented according to their ranking and score during prioritization. Cell line tested and dose and time of treatment applied to each cell line are demonstrated in separate columns.

Disease	Rank	Score	Perturbation (Drug)	Cell Line	Dose	Time
FSGS	7	0.0323	PD-0325901	HEPG2	10.0 um	24 h
MCD	1	0.0567		HT29	0.12 um	24 h
IgAN	45	0.0243		A375	1.11 um	24 h
TBNM	36	0.0213		HT29	0.12 um	24 h
MN	5	0.0192		HT29	0.12 um	24 h
FSGS	9	0.0301	PD-184352	A375	3.33 um	24 h
MCD	7	0.0482		HT29	10.0 um	24 h
IgAN	16	0.0273		HT29	10.0 um	24 h
TBNM	27	0.0218		HT29	10.0 um	24 h
MN	12	0.0170		HT29	10.0 um	24 h
FSGS	44	0.0237	KU 0060648 trihydrochloride	A375	10.0 um	24 h
MCD	20	0.0425		A375	10.0 um	24 h
IgAN	24	0.0258		A375	10.0 um	24 h
TBNM	8	0.0257		MCF7	10.0 um	24 h
MN	20	0.0157		A375	10.0 um	24 h

**Table 3:** Potential mechanism of action, target disease, side effects and FDA approval of drugs and small molecules. MOA: mechanism of action; N/A: not available.

Perturbation (Drug)	Mechanism of Action (MOA)	FDA Approval	Disease Target	Side Effects
Tanespimycin	HSP inhibitor	Yes	Multiple myeloma	N/A
JAK3 Inhibitor VI	Unknown	No	Unknown	N/A
BRD-A17065207	Protein synthesis inhibitor	No	Unknown	N/A
Ponatinib	Bcr-Abl kinase inhibitor	Yes	Chronic myeloid leukemia	Hypertension
	FLT3 inhibitor		Acute lymphoblastic leukemia	Rash
	PDGFR tyrosine kinase receptor inhibitor			Abdominal pain
				Fatigue
				Headache
				Dry skin
				Constipation
				Arthralgia
				Nausea
				Pyrexia
				Thrombocytopenia
				Anemia
				Neutropenia
				Lymphopenia
				Leukopenia
NVP-AUY922	HSP inhibitor	No	Unknown	N/A
BRD-K53414658	VEGFR inhibitor	No	Unknown	N/A
KIN001-265	Unknown	No	Unknown	N/A
CGP-60474	Unknown	No	Unknown	N/A
GSK-1059615	Unknown	No	Unknown	N/A
AZD-5438	CDK inhibitor	No	Unknown	N/A
GSK-2126458	Unknown	No	Unknown	N/A
AZ-628	RAF inhibitor	No	Unknown	N/A
Z-Leu3-VS	Unknown	No	Unknown	N/A
CGP 71683 hydrochloride	Neuropeptide receptor antagonist	No	Unknown	N/A
Ketorolac TROMETHAMINE	Cyclooxygenase inhibitor	Yes	Unknown	Abdominal pain
				Dyspepsia
				Nausea
				Headaches
				Vomiting
				Epigastric pain
				Gastrointestinal bleeding
				Drowsiness
				Acute renal failure
				Hypertension
				Respiratory depression
				Coma
16-HYDROXYTRIPTOLIDE	RNA polymerase inhibitor	No	Unknown	N/A
89671	Unknown	No	Unknown	N/A
BRD-K13810148	HDAC inhibitor	No	Unknown	N/A
Pracinostat	HDAC inhibitor	No	Unknown	N/A
Norethindrone	Progesterone receptor agonist	Yes	Menopause	Adverse effects of contraceptives
			Osteoporosis	nausea
			Vaginal atrophy	vomiting
5-Fluorocytosine	Inhibition of RNA and DNA biosynthesis	Yes	Bacterial septicemia	Nausea
			Endocarditis	Diarrhea
			Urinary tract infections	Vomiting
			Meningitis	Abdominal pain
TWS119	Glycogen synthase kinase-3β inhibitor	No	Unknown	N/A
DL-PDMP	Unknown	No	Unknown	N/A
Taxifolin-(+/−)	Opioid receptor antagonist	No	Unknown	N/A
EI-293	Unknown	No	Unknown	N/A
Neratinib	EGFR inhibitor	Yes	Breast cancer	Hepatotoxicity
				Diarrhea
				Vomiting
				Dehydration
				Cellulitis
				Renal failure
				Erysipelas
				Alanine aminotransferase increase
				Aspartate aminotransferase increase
				Nausea
				Fatigue
				Abdominal pain
BMS-754807	IGF-1 inhibitor	No	Unknown	N/A
BRD-K19295594	BCL inhibitor	No	Unknown	N/A
	MCL1 inhibitor			
BRD-K65814004	Nitric oxide synthase inhibitor	No	Unknown	N/A
DG-041	Unknown	No	Unknown	N/A
15-Deoxy-12,14-prostaglandin J2	Natural peroxisome	No	Unknown	N/A
	Proliferator-activated			
	receptor-γ (PPAR-γ) agonist			
Dovitinib	EGFR inhibitor	No	Unknown	N/A
	FGFR inhibitor			
	FLT3 inhibitor			
	PDGFR tyrosine kinase receptor inhibitor			
	VEGFR inhibitor			
Mitoxantrone	Topoisomerase inhibitor	Yes	Multiple sclerosis	Leukopenia with infection
			Prostate cancer	
			Acute myeloid leukemia	
7b-cis	Unknown	No	Unknown	N/A
MK-0591	Leukotriene synthesis inhibitor	No	Unknown	N/A
Piperlongumine (HPLC)	Unknown	No	Unknown	N/A
S1367	Topoisomerase inhibitor	No	Unknown	N/A
NVP-BEZ235	mTOR inhibitor, PI3K inhibitor	No	Unknown	N/A
SB590885	Unknown	No	Unknown	N/A
Foretinib	VEGFR inhibitor	No	Unknown	N/A
WH-4-025	Unknown	No	Unknown	N/A
GDC-0980	mTOR inhibitor, PI3K inhibitor	No	Unknown	N/A
TPCA-1	IKK inhibitor	No	Unknown	N/A
S1175	HSP inhibitor	No	Unknown	N/A
BRD-K41859756	HSP inhibitor	No	Unknown	N/A
Palbociclib	CDK inhibitor	Yes	Breast cancer	Neutropenia
				Leukopenia
				Anemia
				Fatigue
				Nausea
				Diarrhea
				Respiratory infection
				Headache
				Thrombocytopenia
				Vomiting
				Decreased appetite
Erlotinib	EGFR inhibitor	Yes	Non-small-cell lung cancer	Diarrhea
			pancreatic cancer	Rash
				Liver transaminase elevation
Narciclasine	Unknown	No	Unknown	N/A
BRD-A93236127	ATPase inhibitor	No	Unknown	N/A
BRD-A62809825	Unknown	No	Unknown	N/A
Homoharringtonine	Protein synthesis inhibitor	Yes	Chronic myeloid leukemia	Myelosuppression
				Bleeding
				Hyperglycemia
				Fetal harm
ARP 101	Unknown	No	Unknown	N/A
CAY10594	Unknown	No	Unknown	N/A
BRD-K91370081	DNA synthesis inhibitor	No	Unknown	N/A
BRD-K32896438	Unknown	No	Unknown	N/A
BRD-K23478508	ATPase inhibitor	Yes	Congestive heart failure	Nausea
			Atrial fibrillation	Vomiting
				Visual changes
				Arrhythmia
BRD-K53308430	Unknown	No	Unknown	N/A
Wortmannin	PI3K inhibitor	No	Unknown	N/A
BJM-ctd2-9	Androgen receptor antagonist	No	Unknown	N/A
HY-10518	Unknown	No	Unknown	N/A
BRD-K06009608	Unknown	No	Unknown	N/A
LDN-193189	Bone morphogenic protein inhibitor	No	Unknown	N/A
BRD-K92317137	Unknown	No	Unknown	N/A
PK-11195	Benozodiazepine receptor antagonist	No	Unknown	N/A
MST-312	Unknown	No	Unknown	N/A
BRD-K80786583	Unknown	No	Unknown	N/A
BRD-K78122587	T-type calcium channel blocker	No	Unknown	N/A
GDC-0941	PI3K inhibitor	No	Unknown	N/A
PERHEXILINE MALEATE	Unknown	No	Unknown	N/A
Dorsomorphin dihydrochloride	Unknown	No	Unknown	N/A
BRD-U33728988	Unknown	No	Unknown	N/A
BRD-A80502530	Unknown	No	Unknown	N/A
PHA-665752	c-MET inhibitor	No	Unknown	N/A
JW-7-24-1	Unknown	No	Unknown	N/A

## Supplementary Materials

The following are available online at https://www.mdpi.com/article/10.3390/jcm10112262/s1, Table S1: Clinical parameters and demographics of patients and healthy individuals; Table S2: Glomerulonephritis-specific differentially expressed genes; Table S3: Drugs and small-molecule compounds predicted to reverse each primary glomerular disease.
